# Cardiac telerehabilitation as an alternative to centre-based cardiac rehabilitation

**DOI:** 10.1007/s12471-020-01432-y

**Published:** 2020-06-03

**Authors:** R. W. M. Brouwers, H. J. van Exel, J. M. C. van Hal, H. T. Jorstad, E. P. de Kluiver, R. A. Kraaijenhagen, P. M. J. C. Kuijpers, M. R. van der Linde, R. F. Spee, M. Sunamura, N. H. M. K. Uszko-Lencer, T. Vromen, M. E. Wittekoek, H. M. C. Kemps, the Committee for Cardiovascular Prevention and Cardiac Rehabilitation of the Netherlands Society of Cardiology

**Affiliations:** 1 Flow, Centre for Prevention, Telemedicine and Rehabilitation in Chronic Disease, Máxima Medical Centre, Eindhoven, The Netherlands; 2 Basalt Rehabilitation, Leiden, The Netherlands; 3 Department of Cardiology, Slingeland Hospital, Doetinchem, The Netherlands; 4 Department of Cardiology, Amsterdam University Medical Centre, University of Amsterdam, Amsterdam, The Netherlands; 5 Isala Heart Centre, Zwolle, The Netherlands; 6 NDDO Institute for Prevention and E-health Development (NIPED), Amsterdam, The Netherlands; 7 Department of Cardiology, Maastricht University Medical Centre, Maastricht, The Netherlands; 8 Department of Cardiology, Nij Smellinghe Hospital, Drachten, The Netherlands; 9 Department of Cardiology, Máxima Medical Centre, Veldhoven, The Netherlands; 10 Capri Cardiac Rehabilitation, Rotterdam, The Netherlands; 11 Department of Research and Education, Centre of Expertise for Chronic Organ Failure (CIRO+), Horn, The Netherlands; 12 HeartLife Klinieken, Utrecht, The Netherlands

**Keywords:** Cardiac rehabilitation, Cardiac telerehabilitation, Telemonitoring, Coronary artery disease, Chronic heart failure

## Abstract

Multidisciplinary cardiac rehabilitation (CR) reduces morbidity and mortality and increases quality of life in cardiac patients. However, CR utilisation rates are low, and targets for secondary prevention of cardiovascular disease are not met in the majority of patients, indicating that secondary prevention programmes such as CR leave room for improvement. Cardiac telerehabilitation (CTR) may resolve several barriers that impede CR utilisation and sustainability of its effects. In CTR, one or more modules of CR are delivered outside the environment of the hospital or CR centre, using monitoring devices and remote communication with patients. Multidisciplinary CTR is a safe and at least equally (cost-)effective alternative to centre-based CR, and is therefore recommended in a recent addendum to the Dutch multidisciplinary CR guidelines. In this article, we describe the background and core components of this addendum on CTR, and discuss its implications for clinical practice and future perspectives.

## Introduction

In December 2018, an addendum to the Dutch Multidisciplinary Guideline for Cardiac Rehabilitation concerning cardiac telerehabilitation (CTR) was published [1]. In this article, we describe the background and core components of this addendum on CTR, and discuss its implications for clinical practice and future perspectives.

## Background

Multidisciplinary cardiac rehabilitation (CR) reduces morbidity and mortality and increases quality of life in cardiac patients [2–4]. Outpatient CR is a comprehensive intervention, in which patients are offered an individualised centre-based programme that may consist of one or more group-based modules or therapies (i.e. exercise training, education, relaxation therapy, psycho-educative prevention [PEP] therapy, smoking cessation therapy) and/or individual treatment by a psychologist, dietician or social worker [5]. In the Netherlands, the duration of the programme is approximately 12 weeks, and its content is based on an individual assessment of physical, mental, behavioural and social risk factors [6]. Traditionally, CR programmes have mainly been developed for patients with coronary artery disease (CAD), but in the last two decades it has been demonstrated that other cardiac patients benefit from CR as well (Tab. 1; [3, 7]).

**Table 1:** Indications for cardiac rehabilitation

Indications for cardiac rehabilitation
Coronary artery disease
– Acute coronary syndromes
– Coronary revascularisation a. PCI b. CABG
– Chronic coronary syndromes
Chronic heart failure (mainly HFrEF)
Valve surgery for valvular heart disease
Atrial fibrillation
Congenital heart disease
Implantable cardioverter defibrillator implantation
Heart transplantation

*PCI* percutaneous coronary intervention, *CABG* coronary artery bypass grafting, *HFrEF* heart failure with reduced ejection fraction

Despite proven benefits and strong (class IA) guideline recommendations [8–10], less than half of eligible patients with CAD participate in CR, due to both insufficient referral by medical professionals and suboptimal enrolment of patients who are referred [11, 12]. Data from the EUROASPIRE IV survey [12] suggest that in the Netherlands, 72% of patients with an acute coronary syndrome and/or coronary revascularisation were referred for CR, of which 83% ultimately attended a CR programme (resulting in a 60% participation rate). As participation rates in patients with chronic coronary syndromes and/or without coronary revascularisation and patients with chronic heart failure (CHF) or arrhythmias are dramatically lower (1–30%) [13, 14], we may assume these patients are referred even less often. Multiple factors may contribute to low referral and enrolment rates, including physicians’ and patients’ attitudes towards CR and, importantly, lack of capacity at CR centres or hospitals [15].

A second challenge lies in the fact that once enrolled, up to one third of participants prematurely drop out of a CR programme [12, 16]. Determinants of dropout have been evaluated mainly in patients with CAD and include higher age, lower socio-economic status and worse cardiovascular risk profiles [16–18]. Patient-related individual factors (attitudes towards heart disease or healthcare services) and contextual factors (e.g. social support, accessibility of CR programmes) may lead to discontinuation of CR and suggest that patients would benefit from individually tailored CR programmes [19], although convincing evidence on interventions that increase CR adherence is scarce [20]. Finally, besides low CR utilisation and completion rates, targets for secondary prevention of cardiovascular disease are not met in the majority of cardiac patients [21, 22], indicating that secondary prevention programmes, such as CR, leave room for improvement.

CTR may resolve several barriers at patient level, healthcare professional level and (healthcare) system level that hamper the utilisation of CR and sustainability of its effects [15, 23]. Examples of these barriers include transport difficulties (patient level), low physician endorsement of CR (professional level) or limited facilities to provide supervised exercise training (system level). In CTR, one or more therapies of CR are delivered outside the environment of the hospital or CR centre, using monitoring devices and remote communication with patients, preferably using modern communication technology such as internet or video consultation. Individual health data (e.g. heart rate [HR] during exercise, daily physical activity [PA] or nutritional intake) are monitored to enable personalised feedback and education by a healthcare professional [24]. Recent systematic reviews and meta-analyses show that multidisciplinary CTR or exercise-based CTR is a safe and at least equally (cost-)effective alternative to regular, centre-based CR in patients with CAD or CHF (Tab. 2; [24–27]). CTR may also serve as a highly cost-effective addition to centre-based CR, as demonstrated by the Telerehab III trial [28]. In addition, implementation of CTR may reduce healthcare costs due to reduced absenteeism from work and reduced rehospitalisation rates [24, 29]. Therefore, CTR is considered a valuable secondary cardiovascular prevention component by the European Association of Preventive Cardiology and European Society of Cardiology [23, 30, 31].

**Table 2:** Overview of systematic reviews and meta-analyses on cardiac telerehabilitation

Authors	Studies	Patients	Intervention	Control group	Results
Frederix et al. [24]	37 publications (26 RCTs)	CAD CHF Other^a^	Multidisciplinary CTR (multiple CR modules, delivered either separately or combined)	Centre-based CR (10 studies)	↓ Adverse events and hospitalisations ↑ Adherence to physical activity guidelines (as compared to control group)
				*Usual care* (17 studies)	
				No control group, or none described (10 studies)	
Huang et al. [26]	9 RCTs	CAD	Remotely supervised exercise training programme	Centre-based CR (9 RCTs)	No difference in mortality, CV risk factors, QoL (as compared to centre-based CR)
Rawstorn et al. [25]	11 RCTs	CAD	Remotely supervised exercise training programme	Centre-based CR (5 RCTs);	↑ PAL; ↑ EA; ↓ DBP; ↓ LDL‑C (as compared to centre-based CR)
				*Usual care* (6 RCTs)	
Van Veen et al. [27]	19 RCTs	CAD CHF	E‑coaching (online communication)	Centre-based CR (1 RCT)	↑ Functional capacity; ↑ Psychosocial well-being
				Telephone coaching (1 RCT)	
				*Usual care* (17 RCTs)	

*RCT* randomised controlled trial, *CAD* coronary artery disease, *CHF* chronic heart failure, *CR* cardiac rehabilitation, *CTR* cardiac telerehabilitation, *LDL‑C* low-density lipoprotein cholesterol, *CV* cardiovascular, *QoL* quality of life, *PAL* physical activity level, *EA* exercise adherence, *DBP* diastolic blood pressure

^a^Other patient subgroups: after cardiac surgery, implantation of an implantable cardioverter defibrillator or with congenital heart disease. Adapted from [1]

## Patient selection and referral for CTR

Based on the available scientific evidence, two CTR modules are recommended as an alternative or addition to centre-based CR, namely remotely supervised exercise training (teleFIT module) and remotely supervised PEP therapy (telePEP module). In the future, other CR modules (e.g. education, cardiovascular risk management and nutritional counselling) could also be delivered remotely while retaining the multidisciplinary character of the intervention. The choice of either a centre-based or a remotely supervised module should depend on a patient’s preference and his or her motivation to complete either module.

**Fig. 1:**
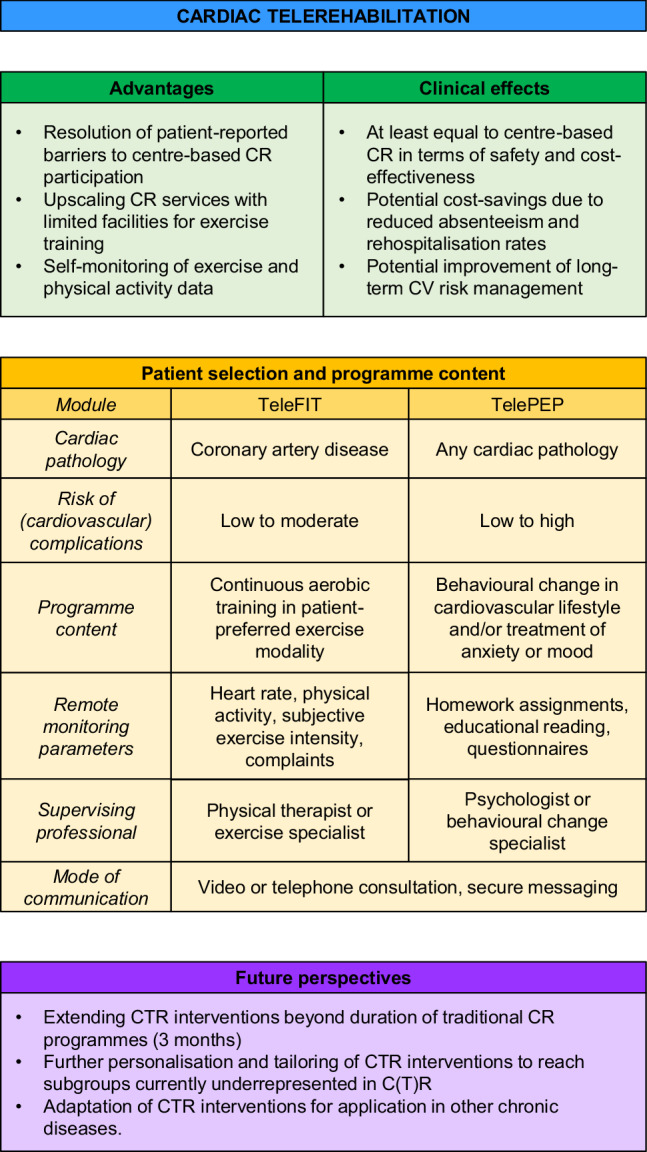
Characteristics of cardiac telerehabilitation programmes. (*CR* cardiac rehabilitation, *CV* cardiovascular, *CTR* cardiac telerehabilitation)

In recent clinical trials evaluating the effects of remotely supervised exercise training as part of CTR (or exercise-based CTR), the majority of patients had CAD (either acute or chronic coronary syndromes) with a low to moderate risk of (cardiovascular) complications [24–26, 32]. Patients with CHF were underrepresented in most exercise-based CTR trials, and only a small number of clinical trials evaluated the effects of exercise-based CTR solely in patients with CHF. Therefore, remotely supervised exercise training as part of CTR is recommended only as an alternative or addition to centre-based CR in low- to moderate-risk (i.e. non-complex) patients [33] with an indication for CR due to any form of CAD (Fig. 1).

In clinical trials evaluating the effects of remotely supervised psychological interventions or interventions to improve lifestyle behaviour, complex patients (mostly with CHF) were better represented than in exercise-based CTR trials [27]. Therefore, remotely supervised PEP therapy (see section ‘TelePEP module’) as part of CTR is, more broadly, recommended as an alternative to centre-based CR in low- to high-risk (i.e. both non-complex and complex) [33] patients with an indication for CR, regardless of underlying cardiac pathology.

### Referral

Patients are referred for CR by their cardiologist after an assessment of risk or the presence of contra-indications for CR. Eligibility for CTR modules is assessed similarly to centre-based CR [6], including an intake procedure, exercise test and questionnaires. We recommend that eligibility assessment and CTR modules are managed by a CR centre equipped with a multidisciplinary CR team (including a cardiologist) to ensure early and accurate communication with qualified professionals. We do not recommend that these procedures are supervised by a general practitioner-led team due to insufficient expertise regarding cardiac pathologies, cardiac pharmacology and CR. It should, however, be possible that parts of the exercise programme are executed at the practice of a certified physical therapist outside the CR centre, (remotely) supervised by a multidisciplinary CR team located at the CR centre.

## TeleFIT module

### Content of the programme and patient selection

The content of a remotely supervised exercise programme depends, similar to a centre-based programme, on patients’ individual goals, preferences and functional capacity. It is recommended that a training or exercise modality is selected based on a patient’s preference, as this increases long-term PA and exercise adherence [34]. We only recommend continuous aerobic training for remotely supervised exercise training, as safety data on remotely supervised high-intensity interval training is currently scarce [35, 36]. Patients with myocardial ischaemia and/or ventricular arrhythmia performing low- to moderate-intensity exercise (documented during exercise testing) should be excluded from remotely supervised exercise training and be referred for centre-based CR (or clinical evaluation, as appropriate).

Before the exercise training sessions are transferred to a patient’s home environment, the patient should follow a limited number of supervised centre-based exercise sessions. In these sessions, patients’ goals and preferred training or exercise modality are discussed, and it is possible to evaluate exercise-related physical or mental complaints. In addition, it is discussed how home-based exercise and PA will be monitored and how remote coaching will be executed.

### Remote monitoring and coaching

A physical therapist (or other healthcare professional) specialised in exercise-based CR and trained in motivational interviewing records a patient’s goals and target HR zone (as a measure for exercise intensity) in the electronic health record and/or in a CTR-specific web application. The target HR zone is based on the current Dutch Multidisciplinary Guideline for CR and the patient’s individual goals [5, 37]. During home-based exercise sessions, the patient should at least monitor his HR by means of a HR monitor (chest strap and/or wrist watch). The HR during home-based exercise sessions should be visualised (e.g. in a graph) in an online web (or mobile) application, and HR data should be accessible for both the patient and the supervising healthcare professional. It is recommended that the exercise modality, subjective exercise intensity (e.g. Borg scale) and exercise-related physical or mental complaints are also recorded. Remote coaching can be executed through the online application (secured messaging) and/or telephone and/or video consultation.

Besides monitoring HR, it is recommended that daily PA is monitored in the online application as well. Objectively measured PA is more reliable than self-reported PA, and for objective measurement of PA accelerometers (uni-, bi- or tri-axial) are preferred to pedometers, since they correlate better with energy expenditure and—in contrast to pedometers—are not designed for a specific exercise modality (e.g. walking) [38–40]. Self-monitoring of exercise and PA data increases levels of self-management and self-care, leading to a more sustainable improvement in healthcare behaviour [41–43].

## TelePEP module

PEP therapy is a structured behavioural change programme, aimed at improving an individual’s lifestyle in order to reduce the risk of recurrent cardiovascular events and/or cardiovascular disease progression [5]. The programme focuses mainly on behavioural change in PA, smoking, alcohol consumption, nutrition and stress, but may also address anxiety, depression, or resumption at work. In PEP, several evidence-based therapies can be used, including cognitive behavioural therapy, acceptance and commitment therapy, relaxation techniques and mindfulness.

### Patient selection

Patients eligible for PEP are also eligible for telePEP. It is recommended that every patient entering CR is screened for anxiety, depression and emotional imbalance using validated questionnaires (i.e. Hospital Anxiety and Depression Scale [HADS], 7‑item Generalised Anxiety Disorder Scale [GAD-7], Patient Health Questionnaire [PHQ-9], *Kwaliteit van Leven bij Hartpatiënten* [KVL-H] and RAND 20-Item Short Form Health Survey [RAND SF-20]) [6]. If any of these screening instruments indicate an increased risk for anxiety or depressive disorders, further assessment by a psychologist or psychiatrist is recommended. Patients with known psychopathology may participate in (tele)PEP only after consultation of a psychologist.

### Content of the programme

Before starting telePEP, patients have an intake appointment (face to face or through video consultation) with a healthcare professional specialised in behavioural change and/or motivational interviewing. When anxiety or depression is suspected, a psychologist should be consulted.

If telePEP treatment sessions focus mainly on behavioural change in PA, smoking, alcohol consumption, nutrition or stress, patients can be treated by a healthcare professional specialised in behavioural change and/or motivational interviewing under supervision of a psychologist. When the treatment sessions focus mainly on anxiety and/or depression, direct guidance by a psychologist should be warranted.

### Remote monitoring and coaching

Monitoring of treatment progression and communication can be performed through an online application (secured messaging) and/or telephone and/or video consultation. The online application may include homework assignments, educational reading and questionnaires to monitor treatment progression. Remote coaching may be combined with face-to-face appointments. Mobile applications (e.g. mindfulness or food diary applications) or wearable devices (e.g. HR monitors or accelerometers) can be utilised to support the behavioural change programme.

## Implications for clinical practice

To our knowledge, the addendum on CTR to the Dutch Multidisciplinary Guideline for Cardiac Rehabilitation [1] is the first CTR guideline worldwide. CTR is a promising alternative to centre-based CR for several reasons. First, it may overcome several barriers that currently limit patient participation in CR. Barriers frequently mentioned by patients are transport difficulties, costs, work or social obligations, or a preference for individual instead of group training. However, whether implementation of CTR actually leads to increased participation levels has yet to be evaluated. Second, implementation of CTR may reduce healthcare and/or socio-economic costs. CTR has proved to be at least as cost-effective as centre-based CR. The FIT@Home trial showed reduced absenteeism from work in patients following CTR compared to centre-based CR [29]. The Telerehab III trial showed reduced rehospitalisation rates in patients following CTR as an addition to centre-based CR, compared to centre-based CR alone [28]. Reductions in healthcare costs may serve as an important argument for widespread implementation of CTR, especially since limited training facilities and budget ceilings at CR centres prevent these centres from delivering centre-based CR to all eligible patients. Third, CTR has the potential to improve long-term cardiovascular risk factor management, as self-monitoring of PA increases levels of self-management and self-care, potentially improving lifestyle in the long term. Some recent studies reported higher PA levels in CTR participants after 6 months as compared with centre-based CR [44, 45], whereas another study reported similar PA levels after 1 year [29]. The 4‑year follow-up of the FIT@Home trial will provide insight into the long-term effectiveness of CTR.

## Future perspectives

Although CTR has been shown to be a valid alternative to centre-based CR, near-future developments may increase its uptake and effectiveness, and improve patient satisfaction. Evidence suggests that after initial improvements in lifestyle behaviour following CR, patients often relapse into unhealthy behaviours [22, 46]. Currently, centre-based CR and CTR programmes consist of 12-week interventions, after which patients are followed-up by their cardiologist and/or general practitioner in (bi-)yearly visits. CTR lends itself well for prolonged (beyond 12 weeks) interventions, in which relapse into unhealthy lifestyle behaviours may be prevented by on-demand coaching, on the condition that such interventions are reimbursed by healthcare insurers. The results of extended centre-based CR programmes have so far been disappointing [47]; CTR may potentially be more effective due to better integration of CR into the daily life of participants, and the addition of self-monitoring of lifestyle behaviour. In addition, insights from the marketing domain on e‑persuasion and personalisation strategies [48] may lead to further personalisation and tailoring of CTR interventions, making them usable for multiple subgroups that are relatively underrepresented in C(T)R (e.g. female and elderly patients, patients with low socio-economic status) [49]. Finally, CTR-like interventions may be implemented for cardiovascular prevention or in other chronic conditions (e.g. diabetes mellitus, cerebrovascular disease) where healthy lifestyle behaviour is essential.

## Conclusion

Multidisciplinary or exercise-based CTR is a safe and cost-effective alternative to centre-based CR in patients with CAD or CHF. Implementation of CTR may lead to increased CR participation levels, improved long-term cardiovascular risk management and, ultimately, reduced healthcare and societal costs. Future adaptations of current CTR interventions will likely further increase their effectiveness and applicability in varied patient populations.
